# Placing Ancient DNA Sequences into Reference Phylogenies

**DOI:** 10.1093/molbev/msac017

**Published:** 2022-01-27

**Authors:** Rui Martiniano, Bianca De Sanctis, Pille Hallast, Richard Durbin

**Affiliations:** 1 Department of Genetics, University of Cambridge, Cambridge, United Kingdom; 2 School of Biological and Environmental Sciences, Liverpool John Moores University, Liverpool, United Kingdom; 3 Department of Zoology, University of Cambridge, Cambridge, United Kingdom; 4 Institute of Biomedicine and Translational Medicine, University of Tartu, Tartu, Estonia; 5 Wellcome Sanger Institute, Hinxton, Cambridge, United Kingdom

**Keywords:** ancient DNA, phylogenetic placement, Y chromosome haplogroups

## Abstract

Joint phylogenetic analysis of ancient DNA (aDNA) with modern phylogenies is hampered by low sequence coverage and post-mortem deamination, often resulting in overconservative or incorrect assignment. We provide a new efficient likelihood-based workflow, pathPhynder, that takes advantage of all the polymorphic sites in the target sequence. This effectively evaluates the number of ancestral and derived alleles present on each branch and reports the most likely placement of an ancient sample in the phylogeny and a haplogroup assignment, together with alternatives and supporting evidence. To illustrate the application of pathPhynder, we show improved Y chromosome assignments for published aDNA sequences, using a newly compiled Y variation data set (120,908 markers from 2,014 samples) that significantly enhances Y haplogroup assignment for low coverage samples. We apply the method to all published male aDNA samples from Africa, giving new insights into ancient migrations and the relationships between ancient and modern populations. The same software can be used to place samples with large amounts of missing data into other large non-recombining phylogenies such as the mitochondrial tree.

## Introduction

The development of high-throughput sequencing methods and their application to archeological remains has dramatically changed our understanding of deep human history. Alongside approaches using autosomal loci, the study of Y chromosomes and mitochondria has provided valuable insights, both because of the resolution of the phylogeny and also because they provide information about sex-biased migrations, kinship, and social systems (Kennett *et al.* 2017; Knipper *et al.* 2017; Furtwängler *et al.* 2020). 

However, there are substantial challenges associated with the analysis of ancient DNA (aDNA) in a phylogenetic context, especially for the Y chromosome because of its larger size (approximately 10 Mb of callable sequence) (Poznik *et al.* 2013) comparatively with the mitochondrion (∼16 kb), as well as its lower coverage. The highly degraded nature of aDNA data, including short fragment size, post-mortem deamination, and high fractions of missing genotypes (Hofreiter *et al.* 2001; Poinar *et al.* 2006; Dabney *et al.* 2013), can lead to errors in variant calling and to incorrect placement of aDNA sequences within a phylogeny (Prüfer *et al.* 2010). In particular, many standard phylogenetic methods require significant overlap of genotypes across samples, which is unfeasible when analyzing a large number of ancient samples simultaneously (Kivisild 2017).

Although there are methods which use likelihoods for the placement of sequences into a preestimated phylogenetic tree, such as pplacer (Matsen *et al.* 2010) and RAxML’s Evolutionary Placement algorithm (Berger *et al.* 2011), these do not take the degraded nature of aDNA into account and can provide erroneous assignments. Furthermore, such likelihood methods do not provide explicit output regarding which or how many SNPs were used for the placement, which is relevant for evaluating the reliability of the results and where on the placement branch the ancient sample diverged. Lastly, they are also computationally expensive when applied to the thousands of samples currently available for analysis (Poznik *et al.* 2016; Hallast *et al.* 2021).

Sequencing of the nonrecombining portion of the Y-chromosome has enabled the rapid and unbiased discovery of new Y-chromosome variants. The International Society of Genetic Genealogy (ISOGG; https://isogg.org/tree/, last accessed March 8, 2020) has been cataloging new informative Y-chromosome variants during the last 15 years, and currently lists approximately 73,000 unique biallelic variants with different levels of confidence. However, curation of new variants is time-consuming and problematic: it can take years until variation from new sequencing studies is added to the ISOGG database and despite major effort a considerable subset of variants only have provisional assignments to specific Y-chromosome lineages, or even contain errors which then need revision.

With these aspects in mind, studies such as Schroeder *et al.* (2015) and Fregel *et al.* (2018) have examined allele status in ancient samples at specific branches of large modern Y-chromosome trees such as from the 1000 Genomes project. By including both novel and known mutations, these studies increased the probability of a given ancient sample having reads overlapping informative branch-defining positions, as noted by Poznik *et al.* (2016). However, no publicly available automated way of doing this exists.

### New Approaches

Here we provide software and an associated workflow, pathPhynder, for integrating aDNA data of variable genomic coverage into present-day phylogenies. To increase its specificity, pathPhynder supports updating and expanding the reference tree and panel of known variants by adding present-day sequences, for example, from newly sequenced diverse populations, to maximize the probability of overlap with sparse aDNA sequences and increase lineage informativeness. Furthermore, pathPhynder also provides a visualization tool which allows inspection of the number of markers in support of or in conflict with assignment to each branch. While handling errors and missing data correctly by working in a likelihood framework, pathPhynder is also computationally efficient, scaling linearly with both sites and samples and taking only a fraction of a second to place a query into a large tree.

The inputs to pathPhynder are a preexisting reference phylogeny in standard newick format, a reference VCF file containing the genotypes of the individuals in the phylogeny, and BAM files of the aDNA query samples mapped against the same reference genome sequence as the phylogeny VCF (or alternatively an already processed VCF file of the query samples).

**Fig. 1.**
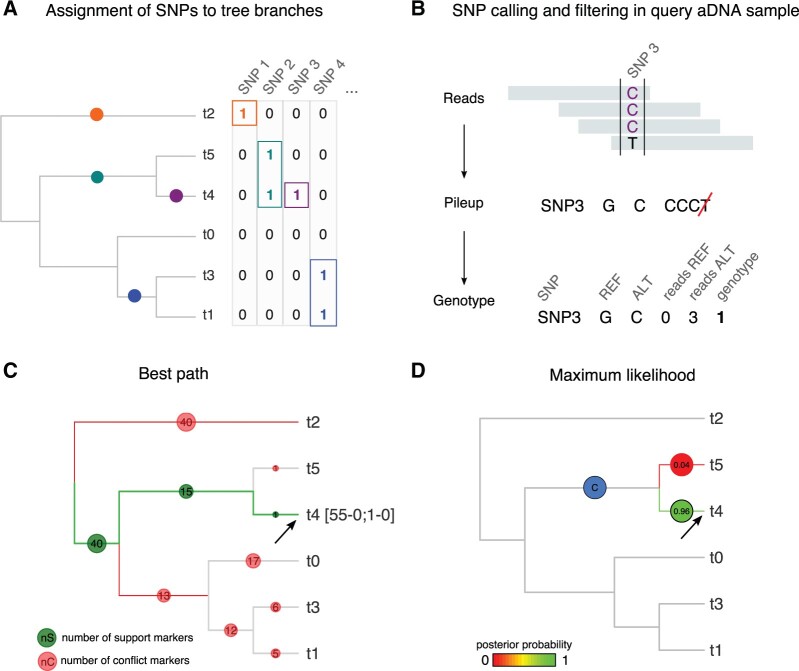
Overview of pathPhynder workflow. We illustrate the method using a small simulated data set of 6 reference samples and 112 SNPs. (*A*) The initial step is the assignment of phylogenetically informative SNPs in the reference data set to branches. This can be achieved with phynder by estimating the likelihood of each SNP at any given branch of the tree. (*B*) A pileup from aDNA reads is generated at each SNP, then filtered for mismatches and potential deamination. Here, because SNP3 is defined by alleles G and C, the T base is excluded as likely to be caused by post-mortem deamination. (*C*) Best path method: aDNA sample genotypes for each SNP are assigned to the corresponding branch of the tree and binned into support and conflict categories. In this case the best path is supported by 56 derived markers (green), of which 55 are above the assigned branch and one is on the branch, with no conflicting markers along the chosen path [55-0; 1-0]. (*D*) Maximum likelihood method: the likelihoods for placing the query sample on each edge of the tree are converted to posterior probabilities using Bayes’ rule and branches with posterior probability greater than 0.01 are indicated (largest posterior in green). The blue circle shows the lowest branch in the tree for which the sum of posterior probabilities for the whole clade below that branch (including the branch in question) is greater than 0.99, providing a conservative assignment when placement is uncertain. The arrows point to the correct location for the query sample.

The pathPhynder workflow is represented in figure 1. The first step assigns informative SNPs from the reference VCF to each branch of the reference phylogeny. This can be achieved by using the “phynder” software, which estimates the likelihood of each biallelic SNP at each branch of the tree. These variants and their location at tree branches can then serve as an initial guide for placing aDNA samples, and for visualization.

Next, a pileup of base calls at the informative sites identified in the previous step is generated for each ancient sample using samtools (by default requiring base quality at least 20 and mapping quality at least 25), and subsequently filtered for error and deamination as follows (fig. 1*B*). First, base calls matching neither the REF nor ALT allele are removed. Then three further filtering modes are available: “no-filter,” where all remaining calls are retained; “default,” in which singleton T calls at C/T sites and singleton A calls at G/A sites are removed to account for possible deamination, and finally, “transversions,” which excludes all transition (C/T and G/A) SNP sites from analysis. Following this, the genotype is called as the most frequent base so long as it is present at least a set fraction (default 70%) of the remaining base calls; otherwise, the genotype is set to missing. An alternative option is to call genotypes at known informative SNP sites using external software and then to pass them to the program as a VCF file.

For the sequence placement step, the user can choose between two distinct methods: best path or maximum likelihood. In the best path method (fig. 1*C*), the SNP counts for a given aDNA sample are assigned to the respective branches, and we traverse possible paths from root to tip in the tree systematically. During this process, if a branch contains a number of conflicting markers greater than a user defined maximum threshold (default 3), the path is stopped and the next one is considered. The path containing the highest number of supporting markers is chosen as the best. This method is akin to the one implemented in yhaplo (Poznik 2016) but applies to all SNPs in the reference samples, rather than just the ISOGG precurated SNP set. The number of mismatches observed at the assigned branch for a given sample is used to estimate where along the branch the sample should be inserted.

The likelihood method (fig. 1*D*) scores the likelihood of placing the query sample on each branch of the tree under a conservative simplifying assumption that ignores mutations on that branch. In addition to identifying the most likely branch, this approach provides Bayesian posterior probabilities for branches with posterior above a user defined threshold *p* (default 0.01) and the lowest branch for which almost all (1 − *P*) of the posterior probability lies on or below the branch thus defining a 99% probability placement clade. Further details are provided in supplementary text S1, Supplementary Material online.

Because the Y-chromosome haplogroup nomenclature is based on the ISOGG data set, we also implemented a procedure to reconcile the reference phylogeny based on a VCF genotype data set with the ISOGG phylogeny based on a marker set, and to further test any ISOGG variants that determine sublineages below the assigned location which are not captured by the reference data set. This allows us to combine the power of testing a more complete variant set with testing variants defining the full ISOGG phylogeny.

The pathPhynder software is available under an open source license at https://github.com/ruidlpm/pathPhynder/. Practical considerations for users of the software are discussed in supplementary text S2, Supplementary Material online.

## Results

### Method Performance

We prepared a Y-chromosome data set of 2,014 individuals from genetically diverse populations with genotypes at a total of 121,966 SNPs (supplementary fig. S1*A*, Supplementary Material online, and Materials and Methods). We built a phylogeny from these samples using RAxML. Phynder assigned 120,908 SNPs (99.13% of the total) in the reference VCF file to the branches of the reference phylogenetic tree. A small number of SNPs (*n* = 1,058) were dropped, either because they were multiallelic (*n* = 631) or because they could not be assigned with confidence (*n* = 427), more precisely because their log likelihood was below a threshold, most likely due to repeated mutation or repeated genotype error. This data set contains more variants than all previously published present-day data for the Y chromosome (Karmin *et al.* 2015; Poznik *et al.* 2016; de Barros Damgaard, Martiniano, *et al.* 2018; Bergström, McCarthy, *et al.* 2020; Hallast *et al.* 2021) (supplementary fig. S1*B*, Supplementary Material online) and includes 90,421 variants (75%) not yet cataloged in ISOGG (2019–2020 version).

**Fig. 2.**
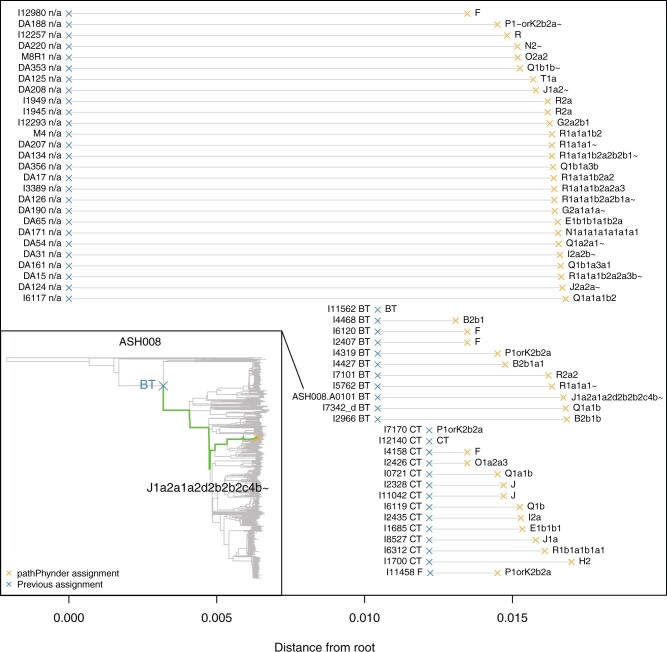
Improvement of Y-chromosome lineage resolution for 52 low coverage samples assigned to higher level branches in the literature. Blue crosses: published assignments. Orange crosses: reassignments by pathPhynder, including ISOGG haplogroup. The phylogenetic tree (inset) provides an example of this process for sample ASH008 (Feldman *et al.* 2019).

To evaluate the advantages of using this additional variation, we selected 52 aDNA samples which had been assigned in the literature using cataloged variants in the ISOGG database to upstream branches of the phylogeny, such as BT-M91 or CT-M168, or were unassigned, and reanalyzed them with pathPhynder. In figure 2, we show the distance between the previous and the newly assigned nodes. In most cases, pathPhynder is able to use additional, uncataloged variation in our new tree to improve the resolution of Y-chromosome lineage assignment (see also supplementary extended table 1, Supplementary Material online).

To compare the resolution of pathPhynder’s haplogroup determination to that of existing software, we processed the same 52 aDNA samples with Yleaf (Ralf *et al.* 2018), yhaplo (Poznik 2016), Y-LineageTracker (Chen *et al.* 2021), and HaploGrouper (Jagadeesan *et al.* 2021) (supplementary extended tables S1 and S2, Supplementary Material online). In most cases, pathPhynder outperforms all other software in both lineage resolution and accuracy by improving the previously published assignment in 94% of cases (*n* = 49/52) and with no incorrectly assigned haplogroups. Haplogrouper showed a similar performance to pathPhynder, improving the assignment of 85% of samples (*n* = 44/52) and achieving higher resolution than pathPhynder in three assignments, but with overall lower resolution that pathPhynder in nine assignments, as well as lower accuracy, with four haplogroups incorrectly determined. Yhaplo had five errors and lower resolution in the majority of the assignments, improving lineage determination in only 65% of the samples (*n* = 34/52), likely due to the fact that it makes use of an outdated ISOGG database (2016) which contains a substantially lower number of SNPs (approximately 20 thousand). Yleaf only improved the lineage resolution for 17 samples (33%) leaving the majority of samples unassigned (56%; *n *= 29/52), and made six incorrect assignments by not taking into consideration a high number of SNPs in the ancestral state leading to the determined haplogroup. Y-LineageTracker had the lowest accuracy of all with a high number of incorrectly determined haplogroups (*n *= 23/52) and improved the lineage assignment in 54% of samples (*n* = 28/52), although the majority of lineages belonged to less resolved haplogroups in upstream branches of the Y-chromosome tree, such as K and P1. In order to account for the underrepresentation of O haplogroup samples in our data set, we expanded our comparative analyses with 12 additional ancient individuals reported in Wang, Yeh, *et al.* (2021) and de Barros Damgaard, Marchi, *et al.* (2018). Our results suggest that both pathPhynder and HaploGrouper perform better than other software, with pathPhynder providing a slightly higher resolution at the haplogroup level than all others, as well as reduced error (supplementary extended table 3, Supplementary Material online).

To examine the impact of coverage on query sample placement and haplogroup determination, we downsampled high coverage ancient (KK1 and BR2) and modern (Bashkir and Uyghur) genomes, which were selected at random, and ran analyses with pathPhynder, Haplogrouper, yhaplo, Yleaf, and Y-LineageTracker (supplementary extended table 4, Supplementary Material online). We observe that pathPhynder provides higher resolution at lower coverages than all the other methods except for Haplogrouper, which provides comparable resolution. According to our analyses, a minimum average coverage of 0.01×, but ideally, 0.03× on the mappable regions of the Y-chromosome is sufficient in many cases to assign haplogroups (supplementary extended table 4, Supplementary Material online). Higher coverage at 0.1-1×, or even more, may be necessary to achieve full resolution.

In further comparisons with RAxML Evolutionary Placement Algorithm (EPA), an existing likelihood-based method for query sample placement in preestimated phylogenies, pathPhynder is comparably accurate in the absence of deamination, but was much more accurate when deamination is present, because of its filtering options (see supplementary text 3 and figs. S2–S5, Supplementary Material online for details). It is also much faster, taking only 1 min and 27 s for placing 30 query samples into our tree with the maximum likelihood option and 16 min and 56 s with the best path option, compared with 76 min and 1 s for RAxML EPA.

### Ancient Y-Chromosome Diversity in Africa

In order to demonstrate the usefulness of our method for real data, we examine ancient and present-day Y-chromosome diversity in Africa by placing all ancient male samples from the African continent published at the time of this study (*n *= 63) (Llorente *et al.* 2015; Schlebusch *et al.* 2017; Schuenemann *et al.* 2017; Skoglund *et al.* 2017; Fregel *et al.* 2018; Van de Loosdrecht *et al.* 2018; Prendergast *et al.* 2019; Lipson *et al.* 2020; Wang *et al.* 2020), and additional samples from the Levant (*n* = 15) (Lazaridis *et al.* 2016) into the Y-chromosome tree using pathPhynder (supplementary table S1, Supplementary Material online).

**Fig. 3.**
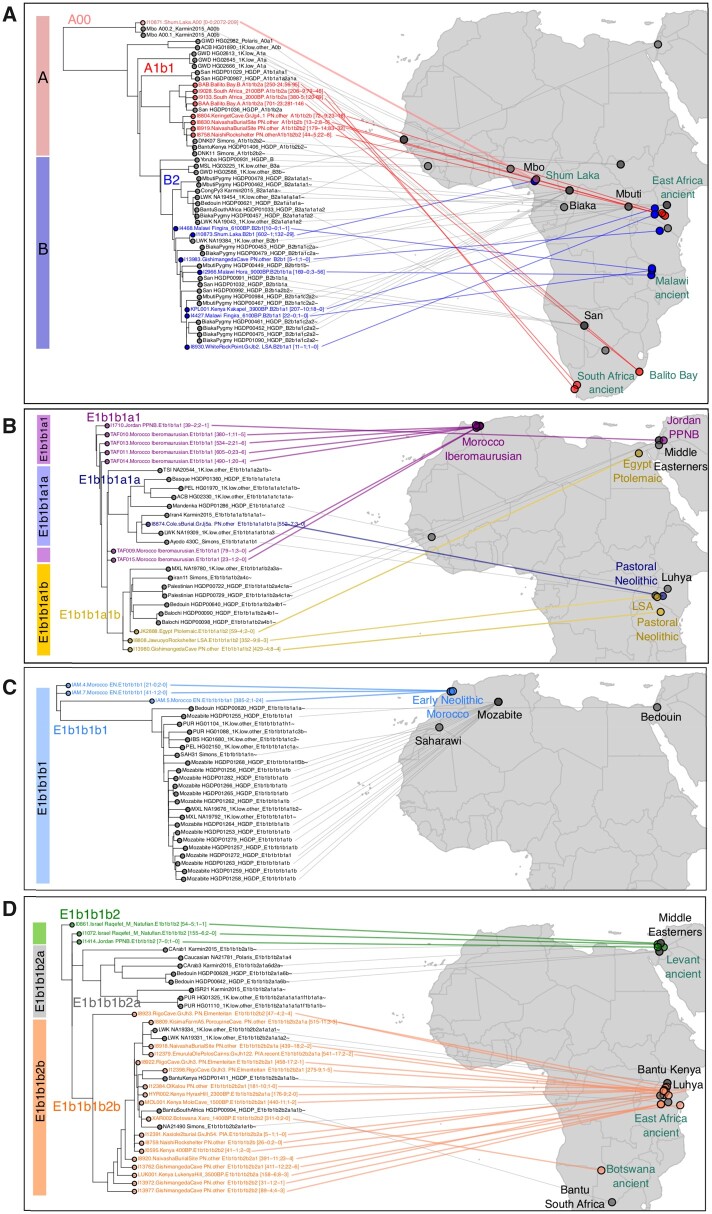
pathPhynder placement of ancient African samples into the Y-chromosome phylogeny. (*A*) A and B lineages, which are mostly composed of present-day San, Mbuti, and Biaka Pygmy populations and ancient hunter-gatherer groups. (*B*) E1b1b1a1 lineages carried by Morocco Ibemaurusian period samples and one Jordan PPNB individual. (*C*) E1b1b1b1 lineages mostly present in Algerian Mozabite populations and shared with Moroccan Early Neolithic samples. (*D*) E1b1b1b2 lineages present in PN samples from East Africa and Levantine Natufians to whom they are ancestrally related.

As expected, the vast majority of the ancient African samples were placed into the A, B, or E clades of the Y-chromosome tree (fig. 3), substantially increasing the lineage resolution of 18 samples (supplementary extended table 5, Supplementary Material online). The most ancestral human Y-chromosome lineage in our data set is A00-L1284 (Mendez *et al.* 2013), which is carried by two Mbo individuals from Western Cameroon (Karmin *et al.* 2015). Here, we establish that these two Mbo individuals in fact belong to the A00b-A4987 lineage. In the aDNA record, a single representative of A00 lineages has recently been identified in Shum Laka Cave, also located in the Cameroon, dating from ∼8 kya (Lipson *et al.* 2020).

pathPhynder placed the Shum Laka sample on the edge leading to the two A00b individuals (fig. 3*A*) with 2072 SNPs supporting this placement and 209 in conflict (including all five of the seven SNPs which ISOGG uses to define the A00b lineage for which Shum Laka has data).

Regarding clade A1-P305, it splits into A1a-M31, present in the Gambian Mandinka, and A1b-P108, present in the South African San and in the Dinka, a Nilotic group from Sudan. The pathPhynder placement revealed a strong geographical pattern: four approximately 2000 year-old South Africans from Balito Bay (Schlebusch *et al.* 2017) and the Western Cape (Skoglund *et al.* 2017) were positioned in the A1b1b2a-M51 clade together with a South African San individual, whereas East African Pastoral Neolithic (PN) samples from Kenya (Keringet Cave and Naivasha burial site) were placed in the A1b1b2b-M13 clade with three present-day individuals, one Kenyan Bantu and two Dinka from Sudan (fig. 3*A*). We note that the Naivasha burial site individual I8830, previously assigned to xBT (probably A) (Prendergast *et al.* 2019), was here assigned to haplogroup A1b1b2b using only variants which are absent from ISOGG, which provides a strong argument for making use of all uncataloged Y-chromosomal variation for increasing the resolution of aDNA phylogenetic placement. The relationships here observed concur with those previously presented based on autosomal variants, with ancient South African individuals being more closely related to the San (Schlebusch *et al.* 2017; Skoglund *et al.* 2017), and Kenyan PN individuals having substantial Dinka-related ancestry (∼40%) with the remaining ancestry coming from North Africa and the Levant (Prendergast *et al.* 2019).

Regarding the B2-M82 lineages, in our data set, these are mostly composed of Biaka, Mbuti, and San individuals. pathPhynder allowed further resolution of the lineage assignment of multiple ancient samples to B2b1-M192, including the second Shum Laka individual who was previously assigned to the B2b-M112 lineage (fig. 3*A*). The reported autosomal affinity of Shum Laka samples with central African hunter-gatherer populations fits with this assignment to the B2 clade. We note, however, that in our data set this sample’s Y chromosome is closest to that of a present-day Luhya individual from Kenya which carries a B2b1 lineage, rather than to those of the sampled Biaka, Mbuti and San.

Three samples from Malawi (two Malawi Fingira_6100BP and one Malawi Hora_9000BP), previously assigned to the BT-M91 macro-haplogroup (Skoglund *et al.* 2017), were in the present analysis further refined to B2b1-M192, B2b1b1a-P6, and B2b1a1-M8349 (fig. 3*A* and supplementary extended table 5, Supplementary Material online). The connection between the Y-chromosome lineages of ancient and present-day South African hunter-gatherer populations corroborates the finding that San-related ancestry was widely distributed in the past, and composed a large part of the ancestry of Malawi hunter-gatherers, previously reported based on autosomal data (Skoglund *et al.* 2017).

Sample Kenya_Kakapel_3900BP which was previously assigned to the CT-M168 clade (Wang *et al.* 2020), was observed to be ancestral to this lineage, and was instead assigned to B2b1a1-M8349, shared with present-day Mbuti and Biaka samples (fig. 3*A*). Fittingly, this sample in particular was observed to share substantial autosomal genetic ancestry with the Mbuti (Wang *et al.* 2020).

The next clade on which we will focus is E1b1b1a1-M78, which has a broad geographical range which encompasses North and East Africa, Europe, and Western Asia (Cruciani *et al.* 2007). All Morocco Iberomaurusian were positioned in this clade (Van de Loosdrecht *et al.* 2018) as well as a Jordan Prepottery Neolithic B (PPNB) individual (fig. 3*B*). A single East African PN sample was placed in the E1b1b1a1a1b-V32 clade, together with a Luo, a Luhya and an Iranian individual. The Egypt Ptolemaic sample, a Late Stone Age and a PN individual were placed in the E1b1b1a1b2-L677 clade, which can be found in the present day in the Horn of Africa and Egypt (Cruciani *et al.* 2007). In our data, this clade is represented by Palestinians, one Bedouin, two Balochi, and one Iranian.

Three Moroccan Early Neolithic samples carry E1b1b1b1-M310.1 lineages (Fregel *et al.* 2018), with the lineage of sample IAM.5 further refined to E1b1b1b1a1 ∼-PF2535 with a single supporting marker. In our analysis, they were placed ancestrally to present-day Mozabite and Saharawi North African lineages (fig. 3*C*), which is in agreement with the finding from autosomal analyses that these samples comprised an endemic Maghrebi element still retained in present-day North African populations (Fregel *et al.* 2018).

Apart from those described above, the majority (*n *= 11) of other East African PN samples were placed in E1b1b1b2b-V1515 lineages (fig. 3*D*), a sub lineage of E1b1b1b2-Z830 found in the Levantine proto-agriculturalist Natufians and a pre-pottery Neolithic B Levantine sample (Lazaridis *et al.* 2016), sister to E1b1b1b2a-Z1145 lineages still found in the Middle East. A subset of those East African PN (*n *= 7) were further assigned to the E1b1b1b2b2a1-M293 lineage, which is a descendant of the Northeast African E1b1b1b2b-V1515 (Trombetta *et al.* 2015) and has been proposed to be associated with the spread of pastoralism from East to South Africa (Henn *et al.* 2008; Prendergast *et al.* 2019). In our data set, this clade is represented by Bantu from Kenya and South Africa, one Maasai and two Luhya individuals from Kenya. Additionally, one early pastoral and two Pastoral Iron Age individuals from Tanzania were placed in the E2a-M41 clade (supplementary fig. S6, Supplementary Material online).

## Discussion

We present the pathPhynder workflow which can efficiently assign informative variants to branches of phylogenetic trees and then use this variation for aDNA sample placement. We demonstrate the utility of our approach by placing aDNA samples into a reference Y-chromosome tree, in many cases leading to increased phylogenetic resolution.

When applying our workflow to place all currently available ancient African male samples into a present-day Y-chromosome phylogeny, we observed patterns of paternal lineage continuity at a regional level as well as evidence for replacement. Samples belonging to ancient hunter-gatherers from Malawi and South Africa were assigned to Y lineages which still persist in present-day South African hunter-gatherers groups. In North Africa, we observe discontinuity between the Y-chromosome lineages carried by 15,000-year-old Iberomaurusian individuals and later Early Neolithic groups who inhabited the region. These Early Neolithic samples from Morocco carried an ancestral lineage to those observed in modern Saharawi and Mozabite populations, suggesting local diversification of these lineages. More extensive sampling of ancient and present-day African groups should reveal more insights about the patterns of Y-chromosomal lineage change and persistence in the region.

Our method works with routinely used formats in aDNA analysis (VCF and BAM files) and does not require alignments in the fasta format, which for large data sets can be computationally expensive and time-consuming to generate.

Our best path option, though a little slower, provides a highly detailed output containing information about the SNPs supporting or in conflict with query sample placement. This is particularly important for aDNA samples because they commonly diverge from the present-day tree at internal branches, in which case they will have a mixture of ancestral and derived genotypes at the SNPs defining this branch. Our visualization tools allow the user to examine this pattern, which is not directly accessible using standard likelihood placement methods.

For Y-chromosome analysis in particular, the majority of aDNA studies rely on a catalog of known haplogroup-defining SNPs maintained by ISOGG, which compiles and curates variation obtained from multiple studies. However, maintaining a curated SNP database inevitably results in a lag between the generation of new data and incorporation of this novel variation into databases. Our method offers an effective solution for immediately making use of uncataloged variation as new data sets emerge, and we provide as a resource the new data set we generated with 90,421 novel assigned markers from 2,014 samples.

On the other hand, in many cases ISOGG SNPs provide additional resolution, illustrated by the fact that 42,863 ISOGG variants are not included in our data set. There are multiple reasons for this: 1) because the 2,014 individuals included in our reference tree fail to capture all the lineages listed in the ISOGG database; 2) we restricted our data set to the ∼10.3-Mb regions of the Y-chromosome where variants can be called unambiguously, as recommended by Poznik et al. (2013). If the same filter is applied to ISOGG, this results in the exclusion of 7,694 SNPs; 3) even within this region some SNPs present in ISOGG may not have been genotyped in the reference VCF file; 4) a small subset of SNPs were not assigned to reference tree branches due to multiple mutations, genotyping error, or possibly inaccuracies in the tree topology, resulting in the presence of the derived allele at unrelated branches of the tree and consequently in low likelihood for SNP assignment and exclusion from analysis.

To address these cases, we added the ability to search for derived ISOGG variants below the branch of initial pathPhynder assignment, and in this way, recover information about the ISOGG sublineages which are absent from our reference data set. As well as integrating additional information in the ISOGG panel with the data from our larger reference data set, this also avoids potential complexities in testing all ISOGG variants, which contain some markers with provisional or uncertain status that can create conflicts.

When comparing pathPhynder with existing haplogroup determination methods, it provides higher accuracy and resolution than others, particularly when dealing with very low coverage aDNA samples, with Haplogrouper showing the most similar, but slightly lower performance. We attribute the higher resolution of pathPhynder to the aDNA-specific filtering applied in our workflow as well as the ability to make use of variants which are absent from the ISOGG database. We also note that none of these methods is specifically tailored for dealing with aDNA sequences.

Our workflow can be applied to any nonrecombining data set, including, but not limited to, the Y-chromosome and the mitochondrial genome, and can also be used for phylogenetic placement of environmental DNA samples into preestimated trees. PathPhynder has already been used to place ancient environmental bear DNA into a mitochondrial phylogeny (Pedersen *et al.* 2021). It is also possible to construct mitochondrial and possibly Y-chromosome trees using exclusively ancient samples, and then use pathPhynder to place additional, lower coverage aDNA data, as recently done for environmental mammoth mtDNA (Wang, Pedersen, *et al.* 2021).

Future applications could include examining ancient Y-chromosome and mitochondrial lineages in ancient cattle (Verdugo *et al.* 2019), wolves, and dogs (Bergström, Frantz, *et al.* 2020; Loog *et al.* 2020), for which large turnovers have occurred.

## Materials and Methods

### Y-Chromosomal Data

Whole-genome sequenced present-day Y-chromosomal data from 1,208 males from Hallast *et al.* (2021) was complemented by two Y-haplogroup A00b samples from Karmin *et al.* (2015), 41 from de Barros Damgaard, Martiniano, *et al.* (2018b), 16 from Wong *et al.* (2017), and 1,071 samples from the low coverage 1000 Genomes Project data set (Poznik *et al.* 2016). These were combined with ten ancient samples from Fu *et al.* (2014), Gamba *et al.* (2014), Lazaridis *et al.* (2014), Jones *et al.* (2015), Llorente *et al.* (2015), de Barros Damgaard, and Martiniano, *et al.* (2018), Sikora *et al.* (2019). Genotype calling and filtering are described in detail in Hallast *et al.* (2021). Additionally, 334 samples from the 1000 Genomes Project were removed due to ≥10% missing data across the ∼10.3 Mb analyzed Y-chromosomal regions. The vcf files of samples mapped to GRCh37 were lifted over to GRCh38 using picard (v2.7.2) (http://broadinstitute.github.io/picard/), followed by merging with the rest of GRCh38-based data using bcftools (v1.8) (Li *et al.* 2009). Modern samples from the de Barros Damgaard, Martiniano, *et al.* (2018) data set were filtered for minimum read depth of 3, whereas no minimum depth filter was applied to the 1000 Genomes Project, Wong et al. 2017 and ancient samples due to lower coverage. Lastly, sites with 5% of missing calls across samples were removed. In the final data set of 2,014 males, a total of 9,832,836 sites remained, including 121,966 variant sites. The maximum likelihood Y-phylogeny including 2,014 samples and 121,966 variant sites was inferred using RAxML (v8.2.10) with the GTRGAMMA substitution model (Stamatakis 2014). A complete list of the individuals in the reference data set is available at Zenodo (DOI: 10.5281/zenodo.4332182).

We downloaded previously cataloged ISOGG variants from https://isogg.org/tree/, as available on 03/08/2020, restricting our analysis to biallelic SNPs. Haplogroup determination in the reference set was done with pathPhynder using the “no-filter” parameter.

### Ancient DNA Query Sample Placement into the Y-Chromosome Reference Tree

In order to place ancient samples into the reference phylogenetic tree, we first assigned variants present in the reference VCF file using phynder. The resulting branch assignments were processed by pathPhynder using the “prepare” step, which prepares bed format files for calling variants in the ancient samples, as well as producing an annotated sites file including information about the haplogroup defining variants (extracted from ISOGG 2019-2020 version), if any, and at which branch they occur.

We then ran pathPhynder’s “pileup and filter” step to generate a pileup using samtools (Li *et al.* 2009) at the informative sites identified with phynder and filtered these with default parameters, that is, requiring at least that 70% of reads support a single genotype (-c 0.7), and filtered the resulting calls using the “default” mode (-m), which removes potentially deaminated variants from analysis.

The next step is “choose Best Path” in which the tree is traversed and query sample genotypes are evaluated in terms of support or conflict with every branch of the tree. The best path containing the highest number of support markers is chosen, as well as the best position in which to place the ancient sample in the tree. This step generates a plot indicating the best path and various tables which show detailed information about SNP and haplogroup status for each ancient sample.

We then add the ancient samples into the tree and produce a newick file and a plot with the reference phylogeny which includes the query sample placements. After each placement using the best path method, a string containing information about the number of markers along the best path is added to the query sample name as follows: [support above—conflict above; support on branch—conflict on branch].

Finally, because not all ISOGG lineages are represented in our sample set, we test ISOGG SNPs that determine lineages below the assigned branch, and report the most specific ISOGG lineage that is supported by a derived SNP, along with potential alternates.

Results were plotted using the R programming language (R Core Team 2013), and the R packages phytools (Revell 2012), ape (Paradis and Schliep 2019), and ggplot2 (Wickham 2016).

### Comparison with Existing Methods for Y-Chromosome Haplogroup Determination

We compared the performance of pathPhynder’s haplogroup determination method in 52 low coverage aDNA samples (Lazaridis *et al.* 2016; Skoglund *et al.* 2017; Mathieson *et al.* 2018; Feldman *et al.* 2019; Flegontov *et al.* 2019; Narasimhan *et al.* 2019; Ning *et al.* 2019) using available software designed to this purpose: Yleaf v2.2 (Ralf *et al.* 2018), yhaplo v1.1.2 (Poznik 2016), Y-LineageTracker v1.3.0 (Chen *et al.* 2021), and HaploGrouper (Jagadeesan *et al.* 2021). Similarly to pathPhynder, Yleaf can determine haplogroups directly from BAM files, and we ran this analysis by using the parameters -q1 -b1 -r0. Y-LineageTracker also has this ability, however, when applied to low coverage aDNA alignments, it states that no male sample is left for analysis. To circumvent this issue, we resorted to generating a vcf file by calling 73,350 ISOGG variants with bcftools v1.8 (Li *et al.* 2009), first by generating a pileup (–min-BQ 20 and disabling base quality calibration), and using the output to call genotypes with bcftools call (–multiallelic-caller and –ploidy 1). The resulting vcf file was used as an input for haplogroup determination with Y-LineageTracker “classify,” yhaplo and HaploGrouper, which was done with default parameters.

## Supplementary Material

Supplementary data are available at *Molecular Biology and Evolution* online.

## Supplementary Material

msac017_Supplementary_Data
