# Supplementary material for: Placing Ancient DNA Sequences into Reference Phylogenies
Source: Mol Biol Evol. 2022 Jan 27;39(2):msac017. doi: 10.1093/molbev/msac017 (PMC8857924; doi:10.1093/molbev/msac017)
Supplement: msac017_Supplementary_Data [file msac017_supplementary_data.zip › 211228.placing_aDNA_SM.pdf]

Supplementary Information - Placing ancient DNA sequences into reference phylogenies

Rui Martiniano[1,2]*, Bianca De Sanctis[1,3], Pille Hallast[4,5], Richard Durbin[1,5]**

Authors and Affiliations

1. Department of Genetics, University of Cambridge, Cambridge CB2 3EJ, UK.
2. School of Biological and Environmental Sciences, Liverpool John Moores University, Liverpool L3 3AF, UK.
3. Department of Zoology, University of Cambridge, Cambridge CB2 3EJ, UK
4. Institute of Biomedicine and Translational Medicine, University of Tartu, 50411 Tartu, Estonia.
5. Wellcome Sanger Institute, Cambridge CB10 1SA, UK.

Correspondence to *rm890@cam.ac.uk and **rd109@cam.ac.uk.

Supplementary Text 1 - Likelihood-based placement of query sequences into fixed phylogenetic trees

1. Overview

Starting with a fixed phylogenetic tree, or a 'reference tree', the main objective of the method is to place a new sample, which we call the 'query', into the tree without changing the original topology, using sequence data such as mapped ancient DNA reads. To do this, we use a likelihood approach to calculate the probability of the query attaching at each edge. We do not, however, compute the length of the new branch on which the query lies. This length would depend almost exclusively on the sites differing in the query from all those on the existing tree, which are relatively vulnerable to noise in the data. Since our algorithm is designed primarily for ancient DNA, which has higher levels of sequence noise, we do not attempt to ascertain private variants.

The input to the algorithm is a phylogenetic tree in the form of a .newick file, a VCF file containing genotype data for all the tips in the tree and a second VCF file, containing the genotypes of the query samples to be placed in the tree. A query sample VCF file can be prepared from BAM files using pathPhynder. The output is the best branch assignment and, for suboptimal branch assignments within a specified likelihood ratio of the optimum, their respective relative likelihood scores and posterior probabilities under a uniform branch prior. We also optionally report the lowest branch in the tree for which the posterior probability of being assigned to that branch or one below it is greater than $(1 - p)$, where p is a user defined threshold.

2. Likelihood calculation

Ideally, we would like to be able to test the placement of the query at every possible location on each edge. However, since edges are continuous, this is an infinite space and even a discrete approximation on each edge is computationally costly in large trees. We avoid this complexity by an approximation equivalent to assuming that there are no mutations on the edge where the query attaches, so that the position of the query on the edge is irrelevant. For mutations that are indeed on this edge, this approximation corresponds to pushing them up to just after the parent node if the query contained the mutation, or pushing them down to just before the child node if the query did not contain the mutation. We find this approximation to have negligible effect on the results.

In order to calculate the likelihood L of the tree with the query on a given edge e, we use Felsenstein's pruning algorithm [1]. This is the standard likelihood recursion on trees, also known as belief propagation or message passing in the field of graphical models.

We only consider biallelic sites, although as discussed below we do differentiate between transition sites (C↔T or A↔G), which have a higher mutation rate, and transversion sites. We allow for possible errors in the primary data by setting the likelihood of the data at the tip as 0.999 for the recorded allele, and 0.001 for the alternative allele. For missing data the likelihood at the tip is 1.

For each edge e with length l_e we calculate the probability $p_{\text{flip}}(e)$ that an allele will change over the edge, and the probability $p_{\text{stick}}(e)$ that it will stay the same, as

$$p_{\text{flip}}(e) = (1 - \exp(-\mu l_e))/2$$
$$p_{\text{stick}}(e) = (1 + \exp(-\mu l_e))/2$$

for two mutation rates μ, one for transitions and one for transversions, by default setting the transition rate to 4 times the transversion rate, which is the approximate ratio observed in humans, though this can also

be set by the user as a parameter if desired.

Next we calculate for each edge two likelihoods per site. First, the likelihood $L_{\text{below}}(e,x)$ of the data below edge e conditional on the allele at the bottom of the edge having value x, by applying the recursion up the tree from leaves to the root

$$L_{\text{below}}(e,x) = \left(L_{\text{below}}(e_L,x)p_{\text{stick}}(e_L) + L_{\text{below}}(e_L,\bar{x})p_{\text{flip}}(e_L)\right)\left(L_{\text{below}}(e_R,x)p_{\text{stick}}(e_R) + L_{\text{below}}(e_R,\bar{x})p_{\text{flip}}(e_R)\right)$$

where e_L and e_R are the left and right edges below e, and $\bar{x}$ is the alternative allele to x. Second, the likelihood $L_{\text{above}}(e,x)$ of all the other data at the site on leaves that are not below edge e, conditional on the allele at the top of the edge having value x, via the recursion down the tree from the root

$$L_{\text{above}}(e,x) = \left(L_{\text{above}}(e_P,x)p_{\text{stick}}(e_P) + L_{\text{above}}(e_P,\bar{x})p_{\text{flip}}(e_P)\right)\left(L_{\text{below}}(e_S,x)p_{\text{stick}}(e_S) + L_{\text{below}}(e_S,\bar{x})p_{\text{flip}}(e_S)\right)$$

where e_P and e_S are the parent and sibling edges of e, and $L_{\text{above}}(e_P) = 1$ for the edges immediately below the root. Assuming there are no mutations on the edge e as mentioned above, we can then calculate the likelihood of both the top and bottom of the edge e having value x as

$$L(e,x) = L_{\text{above}}(e,x)L_{\text{below}}(e,x)$$

These calculations are all carried out in log space. We then add log likelihoods across sites to determine the log likelihood of the tree with the query placed on edge e. We store site patterns so that if two sites have the same pattern of data in their leaves, we only do the likelihood calculations once.

The method does not require the assignment of variants to edges, but sometimes these hard assignments are desired output. Variant assignment is therefore determined as follows. If there were indeed a mutation on the edge e from x to $\bar{x}$, we would calculate the likelihood $L(e,x)$ as

$$L(e,x) = L_{\text{above}}(e,x)L_{\text{below}}(e,\bar{x})$$

We can then choose the variant assignment based on maximum likelihood. A variant may be assigned to the root edges, in which case it will have equal likelihood of belonging on either one. To make a decision in these cases, we determine the maximum likelihood ancestral node state and then assign the variant to the appropriate branch.

Although we have described these algorithms for rooted trees, the code also calculates likelihoods correctly for unrooted trees which can be represented in .newick files via a “root” node with three descendants.

Optionally we can force the tree to be ultrametric, that is, to have edge lengths such that all the tips are the same distance to the root. This is done by balancing the tree, recursively scaling the left and right subtrees of a node so that they both have the same average height, setting this to the mean of their original average heights.

Supplementary Text 2 - Practical considerations for using the pathPhynder workflow

1. Filtering criteria

In this section we discuss the impact of the different filtering criteria that can be applied in the pathPhynder workflow for phylogenetic placement of ancient DNA sequences.

1.1. Pileup read mismatch threshold (-c)

The '-c' parameter 'Pileup read mismatch threshold' is important for calling the correct genotype in cases where reads containing different alleles are present, i.e. where more than one allele is represented in the pileup. For a variant to pass filtering, reads containing the most frequent allele must occur at least at x proportion of the total reads. A mismatch threshold of 1 is the most stringent, 0.5 is the most relaxed. The default threshold is 0.7.

To demonstrate the effect of different pileup read mismatch thresholds in variant calling (-c), we consider the genotyping of sample I10873 [2] which belongs to haplogroup B2b1, at Y-chromosome position 23849641. According to ISOGG, the G->T mutation 23849641 (marker Z13192) defines the H3a1 haplogroup, and therefore, we expect that sample I10873 carries the ancestral allele. However, the I10873 sample presents 8 reads containing the reference allele (REF) and 1 read with the alternate allele (ALT) at this position, and therefore, the most common allele (the REF allele) occurs at a proportion of 0.89. When filtering the raw base calls using a stringent filter of -c 1, which effectively requires that 100% of reads support the reference of the alternate allele for calling a genotype, the program outputs a missing genotype, because of the single mismatching alternate read. When relaxing the mismatch threshold to the default parameter of -c 0.7, or the least stringent parameter of -c 0.5 the genotype is correctly called as REF. We find that the default parameter of -c 0.7 can, in the majority of cases, filter out the majority of mismatches, which may be caused by sequencing errors, and call the correct genotype.

1.2. Filtering mode (-m)

The filtering mode parameter (-m) defines the strategy for dealing with deamination (C/T and G/A changes) in the variant calling process. It can be set to 'no-filter', 'transversions' or the default mode can be used. In the default mode, potentially deaminated bases T (in C/T SNPs) or A in (in G/A SNPs) are excluded if they are supported by a single read. In the 'transversions' mode, only transversions are used for analysis and all transitions (C/T and G/A are automatically excluded. The user can choose not to filter for deamination by selecting the 'no-filter' mode.

To illustrate the impact of the filtering mode on variant calling, we discuss the genotyping of sample I10873 at Y-chromosome position 6854850 (ISOGG marker Z36744), which defines haplogroup E1b1b1a1a2a1ã(C->T).

Again here, sample I10873, which belongs to haplogroup B2b1, is expected to carry the ancestral allele C at this SNP site. However, a single read containing the alternate allele T is present, most likely as a result of deamination. In this example, if the raw base calls were not filtered (-m no-filter), this sample would be erroneously genotyped as carrying alternate T allele, which would support a derived state at the E1b1b1a1a2a1a defining marker Z36744. When using the default parameter, the single read carrying the T allele is excluded, resulting in a missing genotype. When considering transversions only (-m transversions), the genotype is also set as missing, together with all other transition SNPs in the sample.

The default parameter is appropriate for most applications, effectively removing deaminated bases, which usually (but not always) do not exceed a single read per SNP site. This parameter is particularly useful for non-UDG treated samples, which contain higher rates of deamination, or even partial UDG treated samples, where some deamination persists at the ends of reads. The transversions parameter provides the most stringent type of filtering, removing all transitions from analysis, and can be used as a sanity check to ensure

that the sample assignment is not being affected by deamination in non-enzymatically treated samples. The no-filter mode is most appropriate for cases where deamination is not a concern, such as UDG treated samples and present-day individuals.

1.3. Maximum tolerance threshold (-t)

The maximum tolerance threshold parameter (-t) sets the maximum number of ‘conflict’ markers tolerated in the ancient sample at a given branch of the tree. If the number of conflict markers exceeds the -t parameter (by default 3), the program will stop traversing the tree. This parameter is only relevant to pathPhynder ‘best path’ method, which as explained in the main paper, traverses all tree branches, evaluating the number of support and conflict markers, and not to the phynder ‘maximum likelihood’ method, which works in an entirely different way.

This parameter is important for two reasons: first, by stopping the tree traversal process at branches with a high number of conflict variants, it avoids evaluating membership to every single branch in the tree, speeding up the computation. Second, by tolerating a user-defined number of conflict variants, it allows placing samples in branches for which they only have incomplete membership.

In certain circumstances, especially in very old samples which may have highly divergent lineages or samples whose lineage is not represented in the Y-chromosome tree we have made available, the -t parameter should be set to a very high value (for example, -t 1000) to allow for the sample to be placed despite the high number of conflict variants. In the case of the Shum Laka A00 sample (I10871), for example, we observed 2,072 variants which support the assignment to the branch leading to the two A00b Mbo individuals in our dataset and 209 SNPs which are in conflict with this assignment.

For most cases, a threshold of 3 is appropriate, and the user can examine the multiple outputs for the existence of substantial derived markers below the branch of assignment and increase the threshold value if necessary.

2. Considerations about contamination

Another factor which can be important in the analysis of ancient samples is contamination, either from present-day sources or in some cases from there being multiple ancient individuals in the sample. In our view such contamination is best detected by separate approaches dedicated to this purpose, for example looking at X chromosome heterozygosity in males, or mitochondrial DNA variation (see [3] for a more extended discussion). However, there may also be a signal directly arising from the pathPhynder analysis, in which upon examination of the best path obtained for a given ancient sample two or more distinct haplotypes are supported. In this case, if the contamination is from present-day DNA, it is recommended that the user preprocesses their data sets with a tool such as PMDtools [4], which uses a likelihood framework for selecting reads with post-mortem deamination signatures characteristic of ancient DNA.

Supplementary Text 3 - Comparison of pathPhynder with other phylogenetic placement software.

In order to compare the accuracy of phylogenetic placement between pathPhynder and RAxML EPA (Evolutionary Placement Algorithm), we selected three individuals from our dataset belonging to the Biaka, Bedouin and Brahui populations who carry different Y-chromosome lineages (B2b1a1c2a2∼-B26, J1a2a1a2d2b2b2c4b1c3a1a1∼-ZS5599, R1a1a1b2a1a1a1f∼-Y928, respectively). We introduced missing genotypes at proportions ranging from 0.5 to 0.95 of the total data (∼120,000 SNPs), making a total of 30 query samples for placement in the phylogenetic tree. Prior to the query sample placement we excluded the three individuals from the reference tree. We then used pathPhynder’s best path, phynder’s maximum likelihood and RAxML’s EPA to place the query samples into the tree and compared the obtained result with the known true position in the original tree.

All three methods were similarly accurate and were able to place the query samples in the correct tree location between 77%-80% of the time (Supplementary Figure S2), with errors larger than a node distance of one only occurring beyond 85% missingness ($\sim$18,000 SNPs) (Supplementary Figure S3). Interestingly, in the worst case of 95% ($\sim$6,000 SNPs) missing data both likelihood methods (pathPhynder likelihood and RAxML) assigned to the wrong tips of the tree, perhaps due to these being longer and so having a larger prior in the absence of other data, whereas pathPhynder best path is more conservative, assigning to a higher node in the tree that includes the correct tip (Supplementary Figure S4). The 'clade' option of pathPhynder likelihood does essentially the same thing.

These results were unfiltered for deamination because they used present-day data. If we take the 80% missing point and add 10% deamination errors at transitions to simulate ancient DNA, then using the unfiltered data set the performance degrades substantially for likelihood approaches, with mean node error distances increasing to 27.67 (Supplementary Figure S5). With the 'default' filtering, which removes potential deamination, the error returns to 0. With the transversion-only filter it increases again to 3, because too many sites are dropped. The best path approach is much less affected by deamination because it stops traversing the path when it encounters more than 3 variants (default parameter that can be modified by the user) in conflict with query sample membership at a given branch. We note that straightforward application of standard tools such as RAxML will be equivalent to the no-filtering approach, and that a simplistic approach of restricting to only transversion sites leads to loss of valuable information.

Table S1: Ancient African individuals analysed in the present study.

Context	Number of samples	Study
Neolithic Morocco and Iberia	7	Fregel et al., 2018 [5]
Malawi, Tanzania and South Africa	6	Skoglund et al., 2017 [6]
Ptolemaic Egypt	1	Schuenemann et al. 2017 [7]
Later Stone Age to Iron Age Kenya and Tanzania	27	Prendergast et al., 2019 [8]
Pleistocene North Africa	6	van de Loosdrecht et al., 2018 [9]
Late Stone Age to Iron Age Cameroon	2	Lipson et al., 2020 [2]
Stone Age South Africa	2	Schlebusch et al., 2017 [10]
Late Stone Age to Iron Age Kenya, Congo and Botswana	12	Wang et al., 2020 [11]
Late Epipaleolithic to Early Bronze Age Jordan and Israel	15	Lazaridis et al., 2016 [12]
Ethiopia $\sim$2500BC	1	Gallego-Llorente et al., 2015 [13]

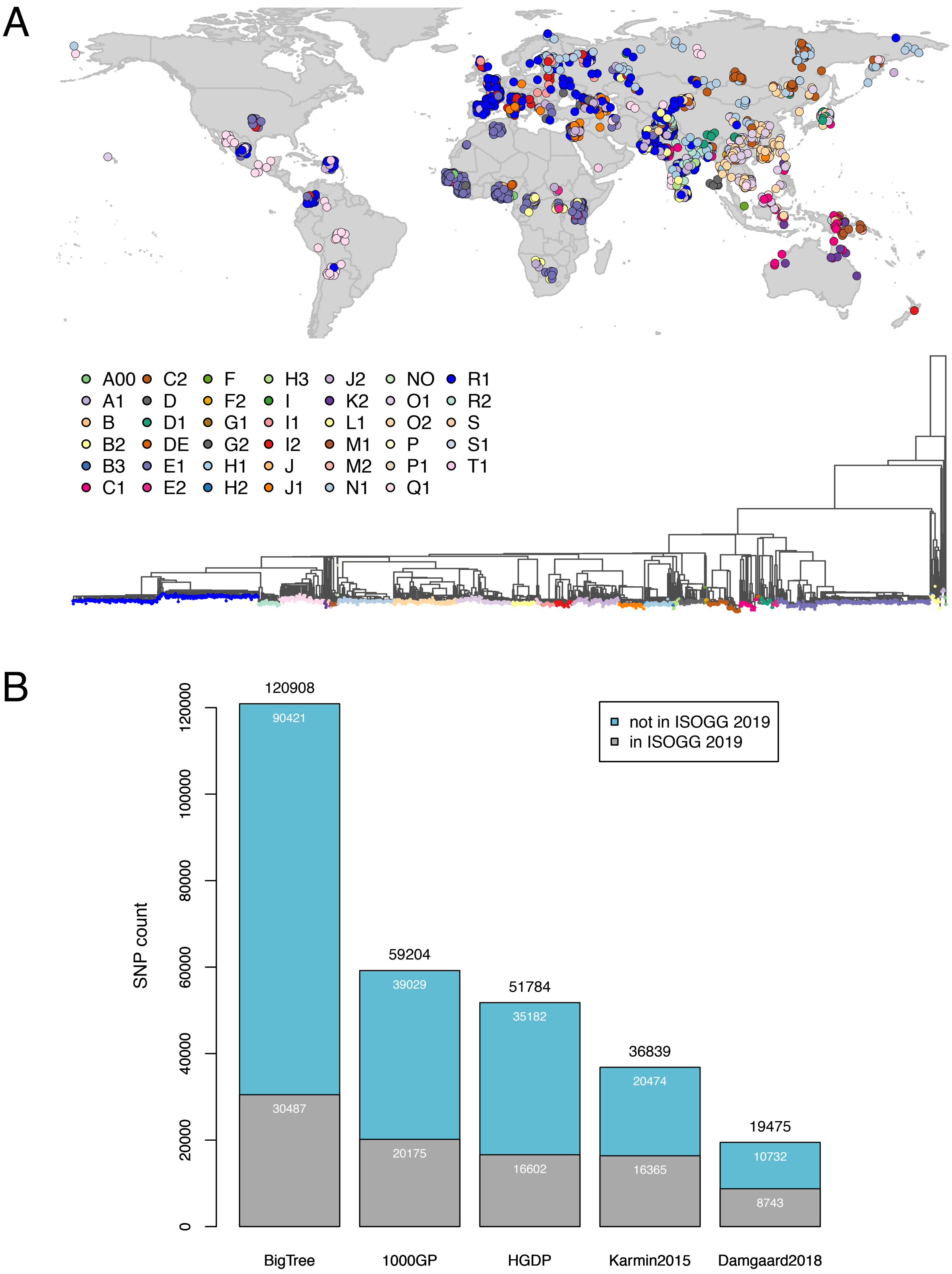

Figure S1: Overview of Y-chromosome SNP variation. A) Phylogenetic tree of the 'BigTree' Y-chromosome reference dataset which we compiled in the present-work and sample location (slightly jittered). B) Total SNP count of different datasets, distinguishing variants that have not yet been included in the ISOGG 2019-2020 database.

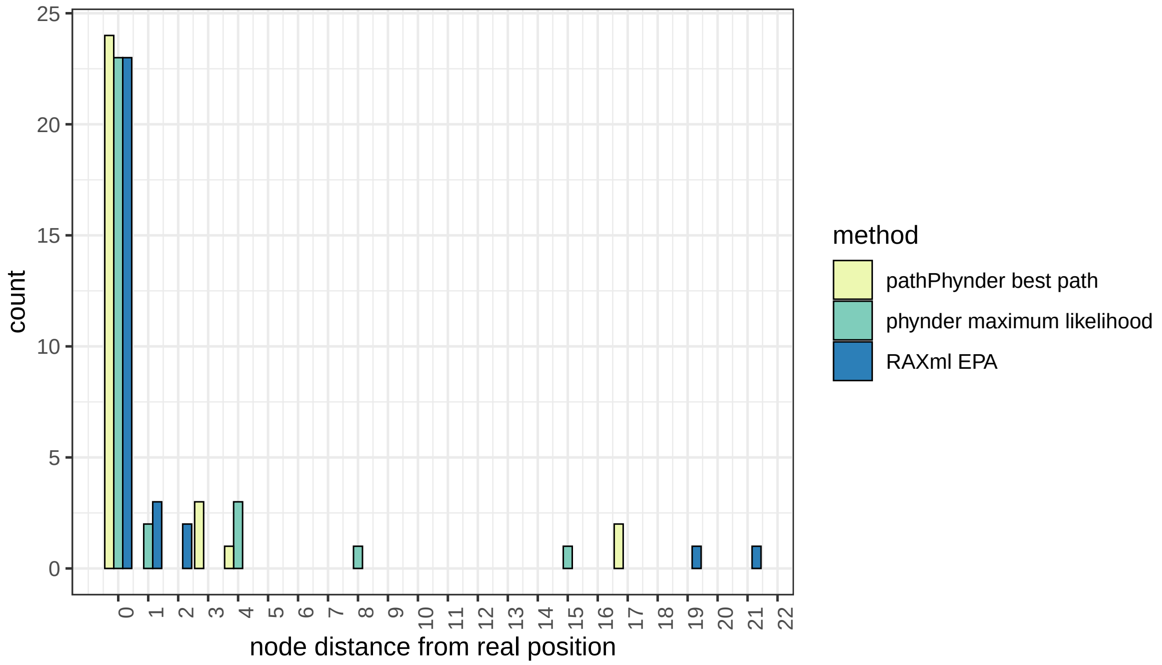

Figure S2: Comparison between three different methods for sequence placement into a reference phylogenetic tree. The x-axis indicates placement distance from the real position measured by the number of nodes which separate them.

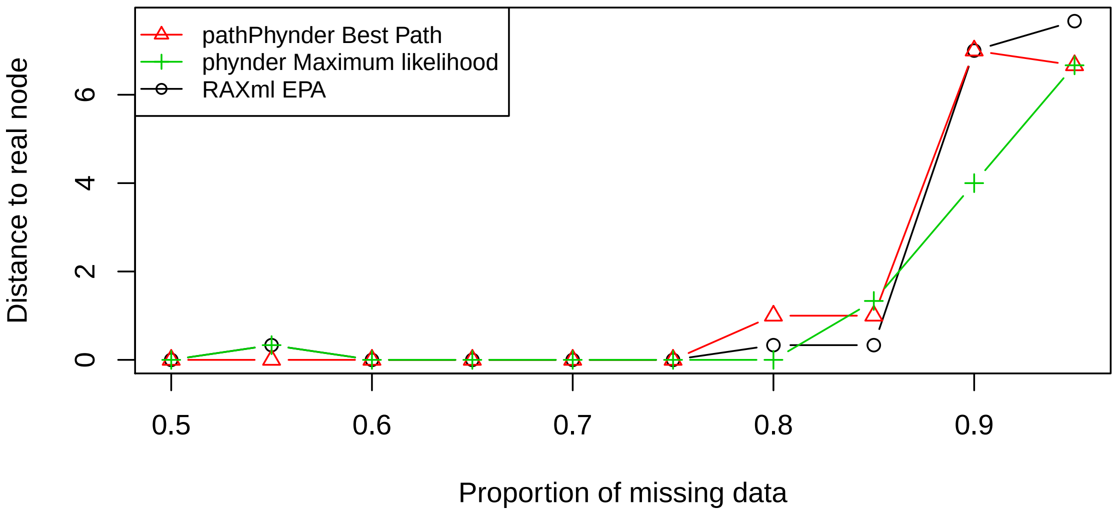

Figure S3: Comparison between three different methods for sequence placement into a reference phylogenetic tree. The x-axis indicates the proportion of missing data and the y-axis shows the mean distance from the real position across the three samples for each downsampling.

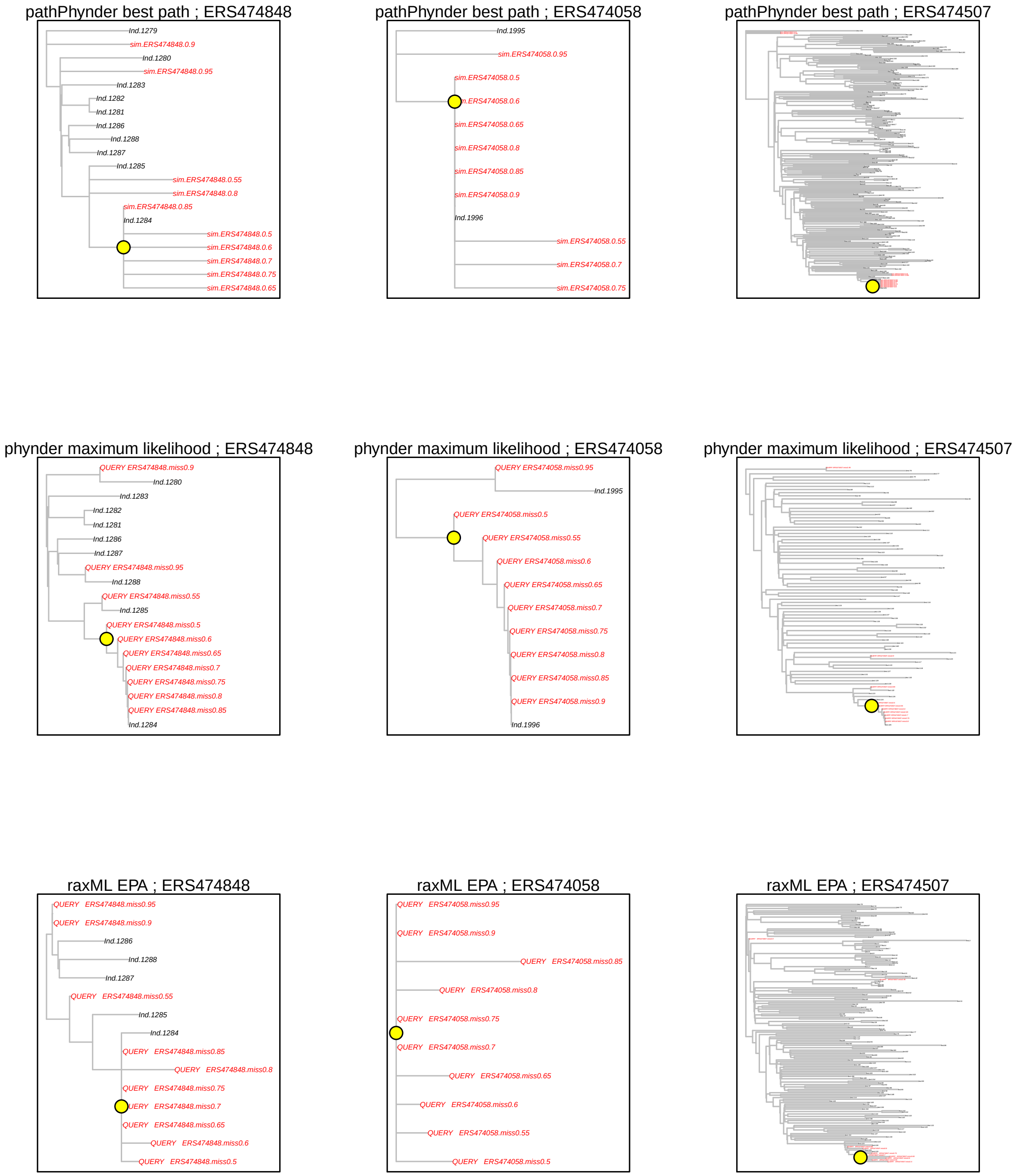

Figure S4: Comparison of the effect of missing data in query placement with RAxML EPA, phynder Maximum Likelihood and pathPhynder Best Path. The query samples are three individuals (ERS474848 - Bedouin, ERS474058 - Biaka, ERS474507 - Brahui) with artificially inserted missing genotypes at a proportion ranging from 0.5 to 0.95, for a total of 30 queries (coloured in red). The yellow circle indicates the correct location in the phylogenetic trees for sample placement.

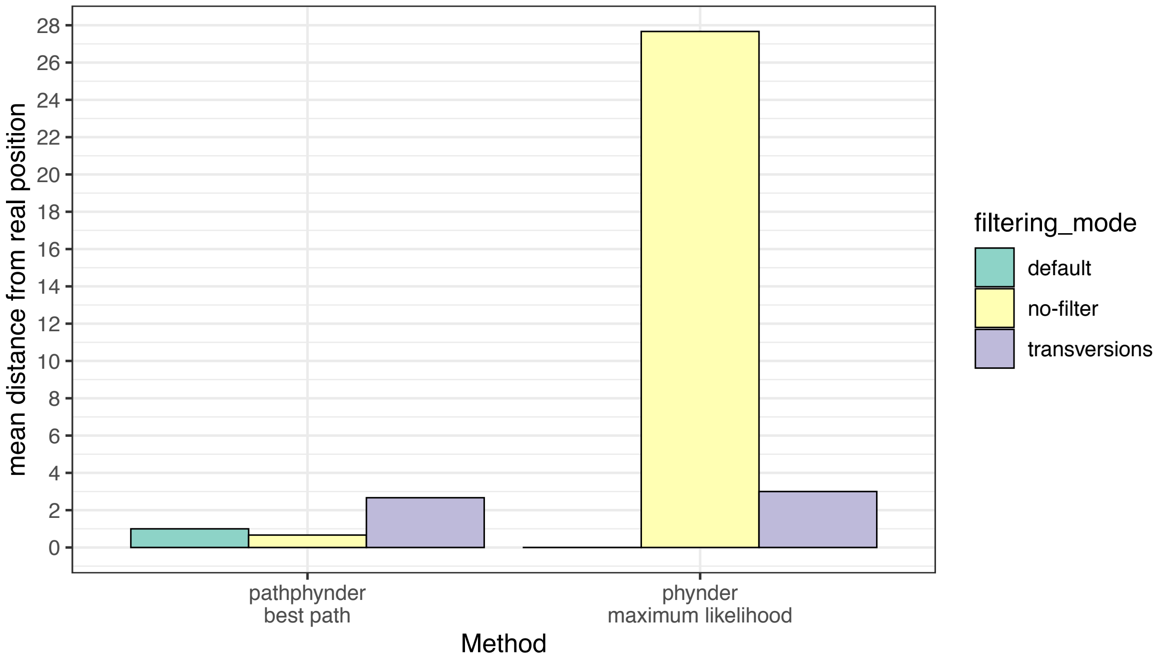

Figure S5: Comparison between the placement of the best path and maximum likelihood methods using different modes for filtering. In the default (conservative) mode, sites with potential deamination are removed, in the no-filter mode, they are kept, and the transversions mode excludes transitions from analysis.

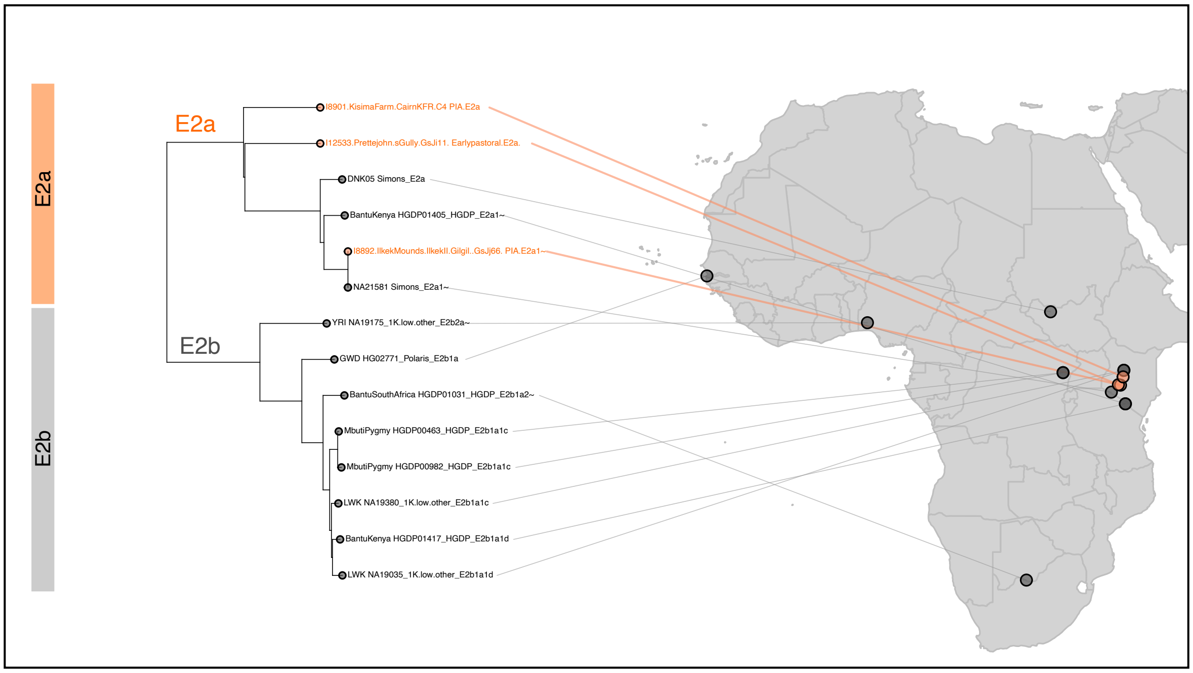

Figure S6: Placement of Early Pastoralist and Pastoralist Iron Age African samples in the E2b clade of the Y-chromosome tree.

References

[1] Joseph Felsenstein. “Evolutionary trees from DNA sequences: a maximum likelihood approach”. In: *Journal of Molecular Evolution* 17.6 (1981), pp. 368–376.

[2] Mark Lipson et al. “Ancient West African foragers in the context of African population history”. In: *Nature* 577.7792 (2020), pp. 665–670.

[3] Stéphane Peyrégne and Kay Prüfer. “Present-Day DNA Contamination in Ancient DNA Datasets”. In: *Bioessays* 42.9 (2020), p. 2000081.

[4] Pontus Skoglund et al. “Separating endogenous ancient DNA from modern day contamination in a Siberian Neandertal”. In: *Proceedings of the National Academy of Sciences* 111.6 (2014), pp. 2229–2234.

[5] Rosa Fregel et al. “Ancient genomes from North Africa evidence prehistoric migrations to the Maghreb from both the Levant and Europe”. In: *Proceedings of the National Academy of Sciences* 115.26 (2018), pp. 6774–6779.

[6] Pontus Skoglund et al. “Reconstructing prehistoric African population structure”. In: *Cell* 171.1 (2017), pp. 59–71.

[7] Verena J Schuenemann et al. “Ancient Egyptian mummy genomes suggest an increase of Sub-Saharan African ancestry in post-Roman periods”. In: *Nature Communications* 8.1 (2017), pp. 1–11.

[8] Mary E Prendergast et al. “Ancient DNA reveals a multistep spread of the first herders into sub-Saharan Africa”. In: *Science* 365.6448 (2019), eaaw6275.

[9] Marieke Van de Loosdrecht et al. “Pleistocene North African genomes link near eastern and sub-Saharan African human populations”. In: *Science* 360.6388 (2018), pp. 548–552.

[10] Carina M Schlebusch et al. “Southern African ancient genomes estimate modern human divergence to 350,000 to 260,000 years ago”. In: *Science* 358.6363 (2017), pp. 652–655.

[11] Ke Wang et al. “Ancient genomes reveal complex patterns of population movement, interaction, and replacement in sub-Saharan Africa”. In: *Science Advances* 6.24 (2020), eaaz0183.

[12] Iosif Lazaridis et al. “Genomic insights into the origin of farming in the ancient Near East”. In: *Nature* 536.7617 (2016), pp. 419–424.

[13] M Gallego Llorente et al. “Ancient Ethiopian genome reveals extensive Eurasian admixture in Eastern Africa”. In: *Science* 350.6262 (2015), pp. 820–822.
